# Bone-specific response according to MDA criteria predicts immunotherapy efficacy among advanced non-small cell lung cancer (NSCLC) patients

**DOI:** 10.1007/s00432-022-04120-z

**Published:** 2022-06-24

**Authors:** Andrea De Giglio, Chiara Deiana, Alessandro Di Federico

**Affiliations:** 1 Department of Experimental, Diagnostic and Specialty Medicine, University of Bologna, Via Giuseppe Massarenti, 9, 40138 Bologna, Italy; 2 Medical Oncology, IRCCS Azienda Ospedaliero-Universitaria di Bologna, Bologna, Italy

**Keywords:** Non-small cell lung cancer, Immunotherapy, Bone metastasis, RECIST criteria, MDA criteria

## Abstract

**Purpose:**

The presence of bone metastasis at baseline has been associated with dismal prognosis under immunotherapy in advanced non-small cell lung cancer (NSCLC). Response Evaluation Criteria in Solid Tumors (RECIST) criteria may be limited for bone-specific response evaluation. Whether their assessment through MD Anderson (MDA) criteria predict immunotherapy efficacy is unknown.

**Materials and methods:**

We conducted a single-center retrospective study to assess the use of MDA criteria in evaluating bone metastasis in NSCLC treated with immunotherapy. Radiological imaging were reviewed to classify bone lesions as osteolytic, osteoblastic, or mixed. Bone response to treatment data was classified according to MDA criteria.

**Results:**

222 patients received single-agent immunotherapy. The presence of bone metastasis increased the risk of death both in the univariate (HR: 1.46, 95% CI, 1.05–2.03, *p* = 0.024) and in the multivariate model (HR: 1.61, 95% CI, 1.10–2.36, *p* = 0.015). According to MDA criteria, 57.3% of patients had progressive disease as best response, 29.5% stable disease, 11.4% partial response and 1.6% complete response. Bone-specific objective response was associated with a significantly increased median overall survival (11.3 vs. 3.1 months, *p* = 0.027) and longer median progression-free survival (6 vs. 2.1 months, *p* = 0.056). The median time to bone failure (TBF) was 2.4 months (IQR, 1.67–3.0). In 25.7% of cases, TBF was shorter than progression-free survival according to RECIST 1.1 criteria. TBF was positively correlated with overall survival (HR = 0.73, *p* = 0.00019).

**Conclusions:**

MDA criteria represent a reliable tool in assessing bone-specific response, offering a more accurate evaluation with the aim to earlier predict survival outcomes or treatment failure compared to RECIST criteria for advanced NSCLC patients receiving immunotherapy.

**Supplementary Information:**

The online version contains supplementary material available at 10.1007/s00432-022-04120-z.

## Introduction

Advanced non-small cell lung cancer (NSCLC) represents the first cause of cancer-related death worldwide (Daniele et al. 2015). In the last decade, the switch from standardized platinum-based chemotherapy toward a biomarker-driven treatment strategy has dramatically extended the life expectancy of NSCLC patients. Programmed cell death-1 (PD-1) and programmed cell death-ligand 1 (PD-L1) inhibitors demonstrated their superiority over chemotherapy in the non-oncogene addicted disease, either as single-agent therapy or combined with chemotherapy, depending on PD-L1 expression (Reck et al. 2016; Mok et al. 2019; Gandhi et al. 2018; Paz-Ares et al. 2018; Di Federico et al. 2021a, b).

Besides PD-L1 expression, other clinical and biomolecular factors, such as the Eastern Cooperative Oncology Group Performance Status (ECOG PS) at diagnosis, the presence of concurrent mutations in specific genes (e.g., STK11 and KEAP1), and location of metastases have been proposed as predictors of response to immunotherapy (Facchinetti et al. 2020; Di Federico et al. 2021a, b; Lindblad et al. 2021). In addition, the tumor microenvironment (TME) may determine different overall or site-specific responses to immunotherapy (Oliver et al. 2018).

Bone represents one of the most frequent metastatic sites of lung malignancies, with an estimated incidence of 30–40% of all patients with NSCLC (Riihimäki et al. 2014). Of all patients with bone lesions, in 60% of cases these metastases are already present at first diagnosis, while in the other 40% they appear in the next 9 months (Daniele et al. 2015). Bone involvement has been associated with poor survival either with platinum-based chemotherapy or immunotherapy in NSCLC patients (Qin et al. 2021; Tournoy et al. 2018; Landi et al. 2019).

Tumor response is widely assessed according to the Response Evaluation Criteria in Solid Tumors (RECIST) (Eisenhauer et al. 2009). However, both RECIST version 1.1 and iRECIST consider bone metastasis as target lesion only in lytic or mixed lytic blastic lesion and with a soft tissue component of at least 10 mm. Therefore, purely osteoblastic or bone lesions with a small soft tissue component cannot be measured with such criteria. Nonetheless, a quality evaluation can be performed for non-target lesions: complete response in case of the disappearance of all lesions and normalization of the tumor marker level, non-complete response or non-progressive disease in case of persistence of one or more non-target lesions and/or presence of tumor marker level above the standard threshold, and progressive disease in case of unequivocal progression or the appearance of new lesions (Eisenhauer et al. 2009; Seymour et al. 2017).

The MD Anderson (MDA) criteria offer a more comprehensive evaluation of bone lesions. The MD Anderson (MDA) criteria offer a more comprehensive evaluation of bone lesions. In fact all bone lesions, including those classified as not target lesions by the RECIST criteria, such as purely osteoblastic lesions and lytic lesions without a soft tissue component, are included in the assessment. Furthermore, what is regarded as response in the MDA criteria includes the disappearance of the lesion for osteoblastic metastases and a qualitative change for lytic lesions, such as the appearance of sclerosis (Hamaoka et al. 2004). Thus, the MDA criteria evaluate bone-specific response to treatment for all patients with bone metastases, as they are designed to include all types of lesions and assess the various changes associated with treatment.

The current work aimed to explore the use of MDA criteria in the evaluation of response in NSCLC patients with bone metastasis. In addition, we investigated whether qualitative differences in bone lesions could affect the response to immunotherapy.

## Materials and methods

We conducted a retrospective, observational study including all consecutive patients affected by advanced NSCLC and treated with single-agent immunotherapy between 2015 and 2021 at the Sant'Orsola-Malpighi University Hospital (Bologna, Italy). We extracted clinical and biological data from medical records. The following variables have been collected: age, gender, tumor histology, smoking status, PD-L1 expression, Eastern Cooperative Oncology Group (ECOG) performance status (PS) at baseline, anticancer treatments, radiological findings at baseline and during the follow-up, last follow-up, cause and date of death.

Two physicians (CD, ADG) independently reviewed radiological imaging of patients presenting bone metastasis at diagnosis, including CT scans and PET with low dose CT scans. Bone lesions were classified as osteolytic, osteoblastic, or mixed-type if both components were present. Bone response to treatments data was collected and classified according to the MDA criteria: osteoblastic lesions were classified as responding to treatment if they decreased in size (PR) or completely disappeared (CR), while lytic lesions were deemed in response if a sclerotic rim appeared (PR) or if they had a complete sclerotic fill-in (CR) (Hamaoka et al. 2004).

**Fig. 1:**
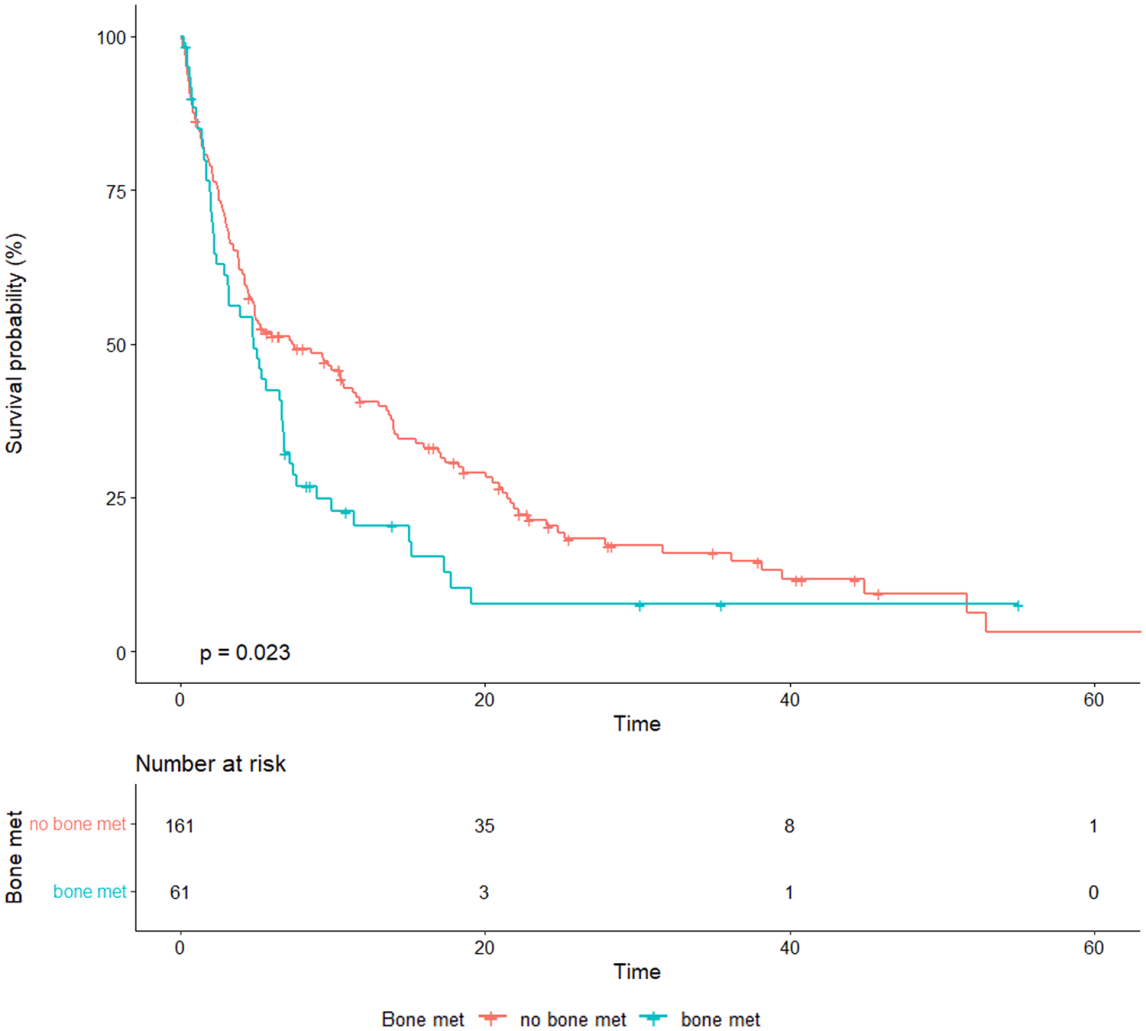
Overall survival according to the presence of baseline bone metastasis in the general population of advanced NSCLC patients treated with single-agent immunotherapy

In Fig. 1S we provided an example of response evaluation according to the MDA criteria.

After appropriate approval from an Internal Independent Ethics Committee (approval no. 2381/2019), we conducted this study following the Declaration of Helsinki (1964).

## Statistical methods

Continuous and categorical variables were described as median values and proportions. T-test (or ANOVA, or Pearson correlation test if needed) and Chi-Squared test (or Fisher's exact test, if needed) were performed to compare means and proportions. Shapiro test was performed to verify the normality of data distribution for each variable of interest.

Overall survival (OS) was defined as the time from treatment start to death from any cause and represented the primary endpoint. Progression-free survival (PFS) was defined as the time occurring from treatment start to the first radiological or clinical disease progression, or death from any cause. Time to bone failure (TBF) was defined as the time occurring from treatment start to first radiological or clinical bone disease progression or death from any cause.

The overall response was defined as a partial or complete response to treatment according to RECIST 1.1 criteria. The bone objective response was described as a partial or complete response of bone lesions to treatment according to RECIST 1.1 or MDA criteria.

Patients still alive at data cut-off (July 2021) were censored at last contact. The Kaplan–Meier method was used to estimate median survival times. The Log Rank Test was used to compare survival outcomes. The reverse Kaplan–Meier method was adopted to calculate the median time of follow-up. A Cox regression model was performed to explore the relationship between clinical or biological variables and survival outcomes. First, a univariate analysis was performed for both survival endpoints; then, variables reaching a *p*-value < 0.1 or considered clinically relevant were included in a multivariable model. A *p*-value ≤ 0.05 was considered statistically significant. Statistical analyses were performed with R-Studio version 1.4.1717, using the following packages: “dplyr”, “prodlim”, “survminer”, “survMisc”.

## Results

### Demographic analysis

A total of 222 patients received single-agent immunotherapy at our institution between March 2015 and June 2021. The median age was 69.5 years (IQR, 63.7–75.1). 61.7% of patients were male, 76.1% had non-squamous histology, 68.1% had a smoking history, and 84% had an ECOG PS of 0 or 1. 50.7% of patients had more than two metastatic sites before the start of immunotherapy. 18.9% and 12.6% of patients showed liver or brain involvement, respectively. 27.5% of patients had ≥ 1 bone metastasis at immunotherapy baseline. Of them, 14.4% were osteolytic, 5.9% were osteoblastic, and 7.2% were mixed-type. Baseline characteristics showed no relevant distribution imbalances, except for a significantly higher prevalence of ≥ 2 metastatic sites among patients with bone metastasis (Table 1).

**Table 1:** Baseline characteristics according to the presence of bone metastasis at baseline

	Patients without bone lesions *N*° (%)	Patients with bone lesions *N*° (%)	Overall population *N*° (%)	*p* value
Age				
≤ 65 years	54 (34.0)	25 (41.7)	79 (36.1)	0.368
> 65 years	105 (66.0)	35 (58.3)	140 (63.9)	
Sex				
Female	64 (39.8)	21 (34.4)	85 (38.3)	0.566
Male	97 (60.2)	40 (65.6)	137 (61.7)	
Histology				
Nonsquamous	122 (75.8)	47 (77.0)	169 (76.1)	0.982
Squamous	39 (24.2)	14 (23.0)	53 (23.9)	
Smoking status				
Former smoker	108 (68.8)	39 (66.1)	147 (68.1)	0.636
Never smoker	19 (12.1)	10 (16.9)	29 (13.4)	
Smoker	30 (19.1)	10 (16.9)	40 (18.5)	
ECOG PS				
0–1	135 (84.4)	49 (83.1)	184 (84.0)	0.977
≥ 2	25 (15.6)	10 (16.9)	35 (16.0)	
PDL-1 expression				
≥ 50%	56 (34.8)	18 (29.5)	74 (33.3)	0.718
0	29 (18.0)	13 (21.3)	42 (18.9)	
1–49%	17 (10.6)	9 (14.8)	26 (11.7)	
Unknown	59 (36.6)	21 (34.4)	80 (36.0)	
No. of metastatic sites				
≤ 2	98 (61.2)	11 (18.0)	109 (49.3)	< 0.001
> 2	62 (38.8)	50 (82.0)	112 (50.7)	
Type of bone met				
No bone met	161 (100.0)		161 (72.5)	< 0.001
Mixed		16 (26.2)	16 (7.2)	
Osteoblastic		13 (21.3)	13 (5.9)	
Osteolytic		32 (52.5)	32 (14.4)	
Liver met				
No	132 (82.0)	48 (78.7)	180 (81.1)	0.713
Yes	29 (18.0)	13 (21.3)	42 (18.9)	
Brain met				
No	141 (87.6)	53 (86.9)	194 (87.4)	1.000
Yes	20 (12.4)	8 (13.1)	28 (12.6)	
Line of treatment				
> 2	26 (16.1)	11 (18.0)	37 (16.7)	0.930
1	44 (27.3)	17 (27.9)	61 (27.5)	
2	91 (56.5)	33 (54.1)	124 (55.9)	
Drug				
Atezolizumab	37 (23.0)	20 (32.8)	57 (25.7)	0.467
Ipilimumab	1 (0.6)		1 (0.5)	
Nivolumab	61 (37.9)	20 (32.8)	81 (36.5)	
Pembrolizumab	62 (38.5)	21 (34.4)	83 (37.4)	

*met.* Metastasis, *SD* standard deviation, *n.* number, *ECOG PS* Eastern Cooperative Oncology Group performance status

### Survival outcomes

Median OS of all 222 analyzed cases was 5.4 months (95% CI, 4.74–7.60). The median time of follow-up was 30.1 months (IQR, 17.95–45.84).

Median OS in patients with bone metastases was 4.8 months (95% CI, 2.86–6.77) versus 7.3 months (95% CI, 4.67–11.51) in patients without bone metastases (*p* = 0.024) (Fig. 1). No survival differences were documented between patients with different types of bone metastasis (*p* for OS = 0.62; *p* for PFS = 0.39).

The presence of bone metastasis was associated with an increased risk of death either in the univariate model (HR: 1.46, 95% CI, 1.05–2.03, *p* = 0.024) or in the multivariate models adjusting for age, histology, number of metastatic sites, line of treatment, PD-L1 expression, brain and liver sites of metastasis (HR: 1.61, 95% CI, 1.10–2.36, *p* = 0.015). Within the same model, the presence of liver metastases at baseline was significantly associated with reduced survival (HR: 1.66, 95% CI, 1.12–2.46, *p* = 0.012) (Table 2).

**Table 2:** Univariate and multivariate analysis for overall survival

	All	HR (univariable)	HR (multivariable)
Age			
≤ 65 years	79 (100.0)	–	–
> 65 years	140 (100.0)	0.97 (0.72–1.32, *p* = 0.858)	1.13 (0.81–1.58, *p* = 0.482)
Histology			
Nonsquamous	169 (100.0)	–	–
Squamous	54 (100.0)	1.21 (0.87–1.69, *p* = 0.263)	1.24 (0.85–1.80, *p* = 0.261)
Line of treatment			
> 2	37 (100.0)	–	–
1	61 (100.0)	0.80 (0.50–1.27, *p* = 0.340)	0.92 (0.43–1.93, *p* = 0.818)
2	125 (100.0)	1.03 (0.70–1.52, *p* = 0.867)	1.08 (0.70–1.65, *p* = 0.734)
No. of metastatic sites			
≤ 2	109 (100.0)	–	–
> 2	112 (100.0)	1.19 (0.88–1.60, *p* = 0.253)	0.90 (0.62–1.32, *p* = 0.594)
PD-L1 expression			
≥ 50%	74 (100.0)	–	–
0	42 (100.0)	1.21 (0.78–1.87, *p* = 0.392)	1.09 (0.56–2.13, *p* = 0.798)
1–49%	26 (100.0)	1.14 (0.67–1.93, *p* = 0.626)	0.92 (0.43–1.96, *p* = 0.823)
Unknown	81 (100.0)	1.33 (0.93–1.89, *p* = 0.120)	1.22 (0.65–2.28, *p* = 0.540)
Brain met			
No	194 (100.0)	–	–
Yes	28 (100.0)	1.56 (1.02–2.40, *p* = 0.040)	1.60 (1.00–2.56, *p* = 0.051)
Liver met			
No	180 (100.0)	–	–
Yes	42 (100.0)	1.51 (1.06–2.17, *p* = 0.024)	1.66 (1.12–2.46, *p* = 0.012)
Bone met			
No	161 (100.0)	–	–
Yes	61 (100.0)	1.46 (1.05–2.03, *p* = 0.024)	1.61 (1.10–2.36, *p* = 0.015)

*HR* hazard ratio, *n.* number., *met.* metastasis

**Fig. 2:**
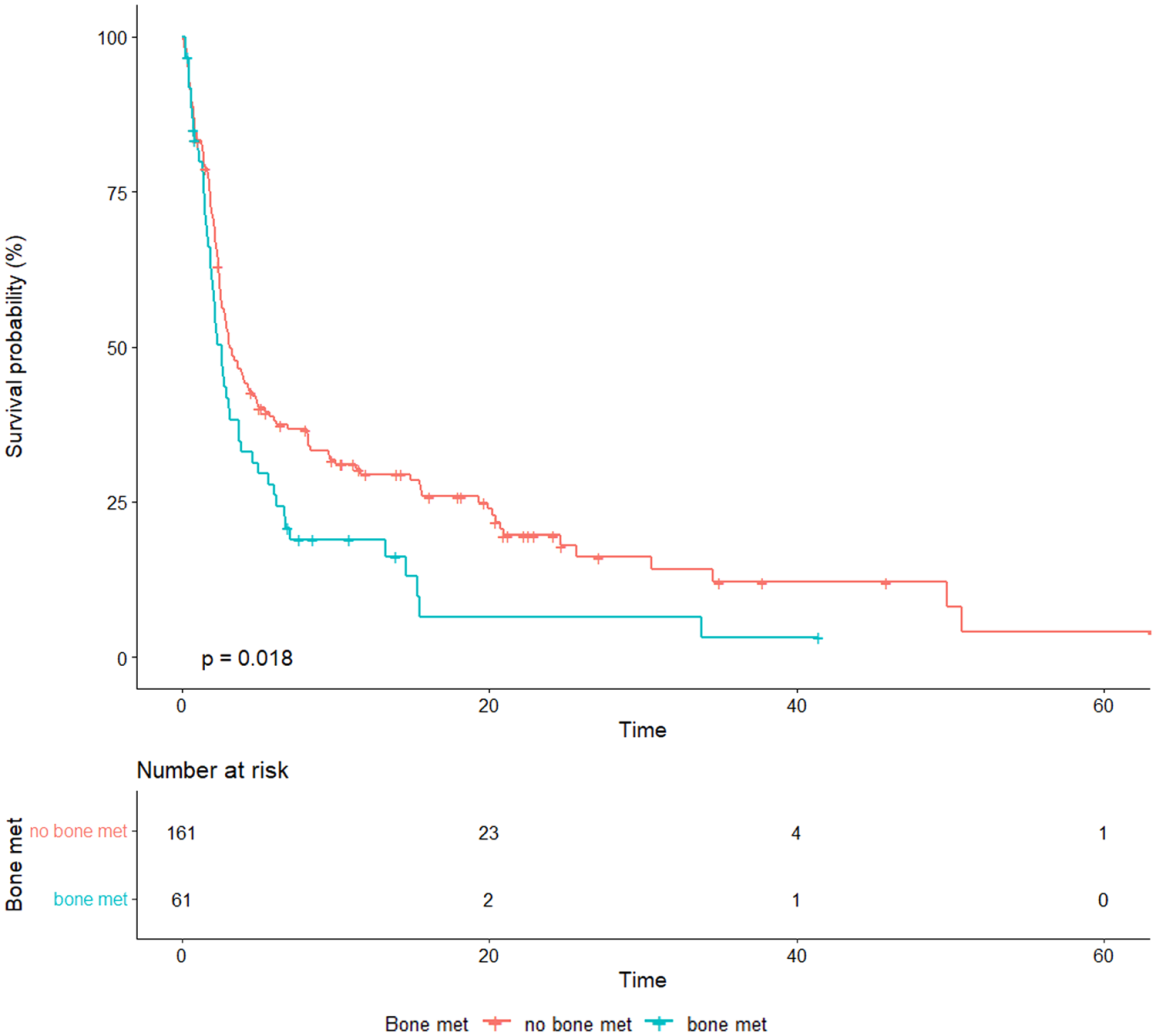
Progression-free survival according to the presence of baseline bone metastasis in the general population of advanced NSCLC patients treated with single-agent immunotherapy

Overall, the median PFS was 2.9 months (95% CI, 2.53–3.85). Patients with bone metastasis experienced a median PFS of 2.5 months (95% CI, 1.91–3.68) versus 3.1 months (95% CI, 2.56–4.83) of those without bone metastasis (*p* = 0.018) (Fig. 2). While the increased risk of disease progression was present in the univariate Cox regression analysis (HR: 1.48, 95% CI, 1.07–2.05, *p* = 0.019), this was not confirmed within the multivariable assessment (HR: 1.42, 95% CI, 0.98–2.08, *p* = 0.067). The presence of liver metastases was confirmed to be significantly associated with disease progression at univariate (HR: 1.49, 95% CI, 1.04–2.15, *p* = 0.031) and multivariate analysis (HR: 1.52, 95% CI, 1.03–2.25, *p* = 0.037). Other variables included in the model, such as age, histology, number of metastatic sites, line of treatment, PD-L1 expression, and cerebral involvement, were not significantly associated with disease progression (Table 3).

**Table 3:** Univariate and multivariate analysis for progression free survival

	All	HR (univariable)	HR (multivariable)
Age			
≤ 65 years	79 (100.0)	–	–
> 65 years	140 (100.0)	0.89 (0.65–1.20, *p* = 0.437)	0.93 (0.67–1.31, *p* = 0.696)
Histology			
Nonsquamous	169 (100.0)	–	–
Squamous	54 (100.0)	1.01 (0.71–1.42, *p* = 0.975)	1.02 (0.69–1.49, *p* = 0.930)
Line of treatment			
> 2	37 (100.0)	–	–
1	61 (100.0)	0.78 (0.49–1.24, *p* = 0.288)	1.06 (0.49–2.29, *p* = 0.888)
2	125 (100.0)	0.98 (0.67–1.46, *p* = 0.938)	1.17 (0.76–1.81, *p* = 0.476)
No. of metastatic sites			
≤ 2	109 (100.0)	–	–
> 2	112 (100.0)	1.31 (0.97–1.76, *p* = 0.074)	1.12 (0.78–1.63, *p* = 0.536)
PD-L1 expression			
≥ 50%	74 (100.0)	–	–
0	42 (100.0)	1.23 (0.80–1.89, *p* = 0.351)	1.14 (0.57–2.27, *p* = 0.704)
1–49%	26 (100.0)	1.15 (0.68–1.94, *p* = 0.614)	0.88 (0.41–1.91, *p* = 0.753)
Unknown	81 (100.0)	1.33 (0.93–1.91, *p* = 0.123)	1.32 (0.69–2.53, *p* = 0.406)
Brain met			
No	194 (100.0)	–	–
Yes	28 (100.0)	1.34 (0.86–2.09, *p* = 0.191)	1.21 (0.75–1.95, *p* = 0.445)
Liver met			
No	180 (100.0)	–	–
Yes	42 (100.0)	1.49 (1.04–2.15, p = 0.031)	1.52 (1.03–2.25, p = 0.037)
Bone met			
No	161 (100.0)	–	–
Yes	61 (100.0)	1.48 (1.07–2.05, *p* = 0.019)	1.42 (0.98–2.08, *p* = 0.067)

*HR* hazard ratio, *n.* number., *met.* metastasis

### Bone specific response according to MDA criteria

**Fig. 3:**
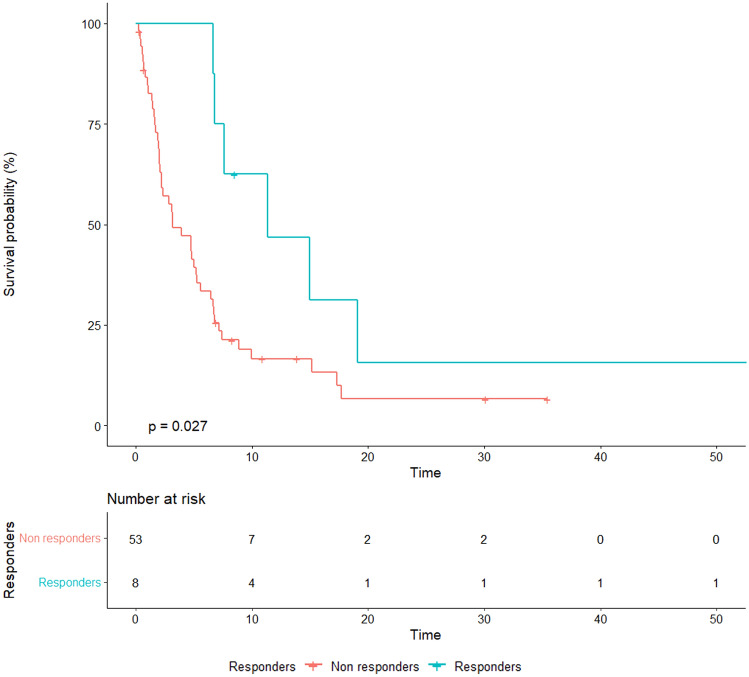
Overall survival according to bone-specific response assessed with MDA criteria

**Fig. 4:**
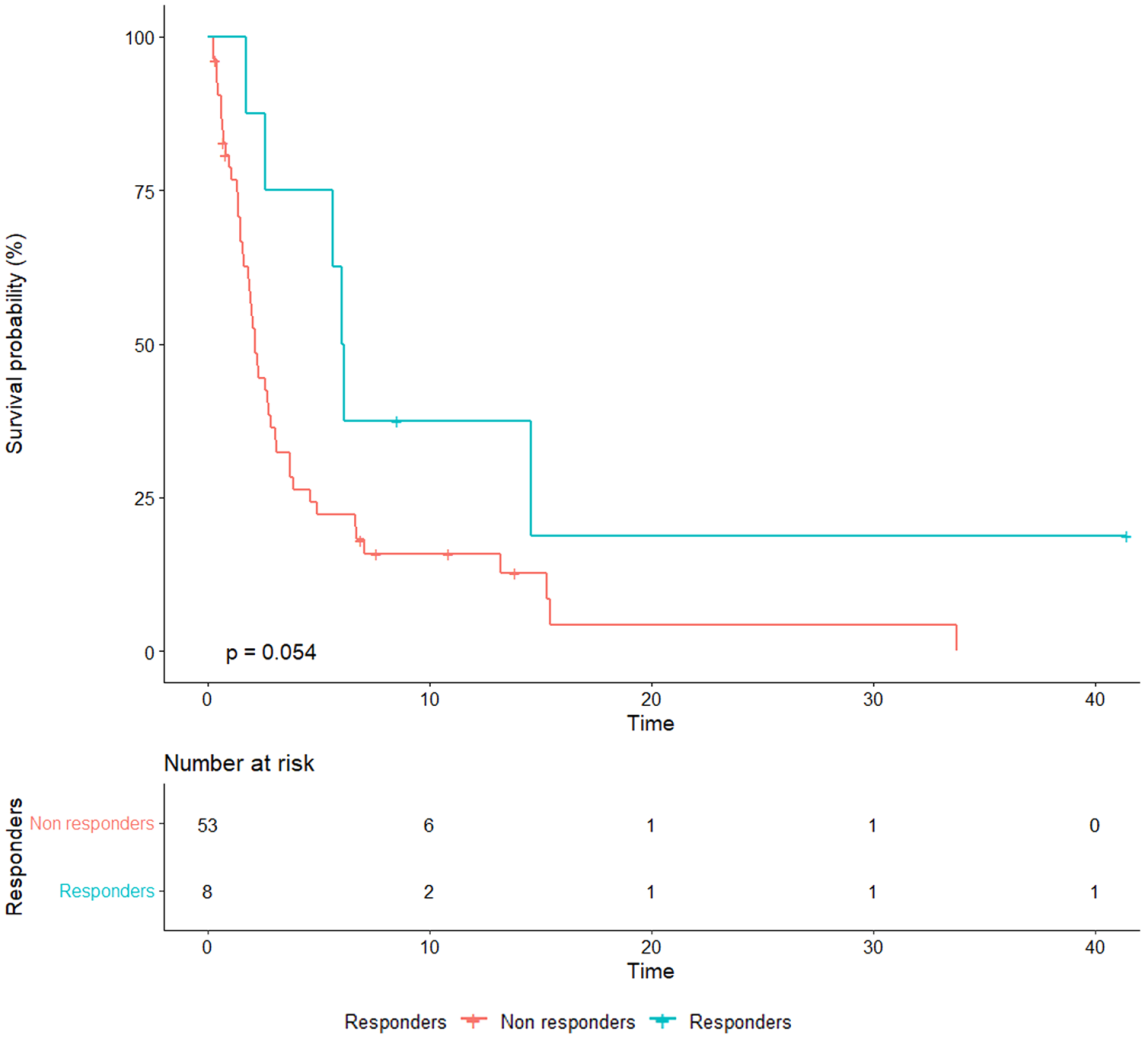
Progression-free survival according to bone-specific response assessed with MDA criteria

According to the MDA criteria, we found that 36 (59%) patients had progressive disease (PD) as best response, 17 (27.8%) stable disease (SD), 7 (11.4%) partial response (PR) and 1 patient (1.6%) had complete response (CR) (Table 4). Bone-specific objective response (PR + CR) was associated with a significantly increased median OS (11.3 vs. 3.1 months, *p* = 0.027) and a trend toward longer median PFS (6 vs. 2.1 months, *p* = 0.056) (Figs. 3, 4).

**Table 4:** Comparative evaluation of bone-specific response through RECIST version 1.1 and MDA criteria

Patient	Recist 1.1 bone lesion evaluation at baseline	Recist 1.1 bone response	MDA bone response	Overall best response
1	Not evaluable	Not evaluable	CR	PR
2	Non-target	Non-CR/Non-PD	SD	PD
3	Non-target	Non-CR/Non-PD	PD	SD
4	Target	PD	PD	PD
5	Non-target	PD	PD	PD
6	Not evaluable	Not evaluable	SD	PD
7	Non-target	Non-CR/Non-PD	PR	PD
8	Non-target	PD	PD	PD
9	Non-target	PD	PD	PD
10	Non-target	Non-CR/Non-PD	SD	PD
11	Non-target	PD	PD	PD
12	Not evaluable	PD	PD	PD
13	Target	PR	PR	PR
14	Not evaluable	PD	PD	PD
15	Not evaluable	PD	PD	PD
16	Not evaluable	Not evaluable	PD	PD
17	Not evaluable	Not evaluable	PD	PD
18	Non-target	PD	PD	PD
19	Not evaluable	Not evaluable	PD	PD
20	Non-target	PD	PD	PD
21	Target	PD	PD	PD
22	Non-target	PD	PD	SD
23	Non-target	Non-CR/Non-PD	SD	PD
24	Non-target	Non-CR/Non-PD	SD	PD
25	Not evaluable	Not evaluable	PD	PD
26	Non-target	PD	PD	PD
27	Not evaluable	PD	PD	PD
28	Not evaluable	Not evaluable	PD	PD
29	Non-target	Non-CR/Non-PD	SD	PD
30	Target	PR	PR	PR
31	Non-target	Non-CR/Non-PD	PR	SD
32	Target	SD	SD	PD
33	Non-target	Non-CR/Non-PD	SD	SD
34	Target	PD	PD	PD
35	Non-target	Non-CR/Non-PD	SD	PD
36	Not evaluable	Not evaluable	PD	SD
37	Non-target	Non-CR/Non-PD	SD	SD
38	Not evaluable	PD	PD	PD
39	Not evaluable	Not evaluable	PD	SD
40	Not evaluable	Not evaluable	PD	PD
41	Not evaluable	Not evaluable	PD	PD
42	Not evaluable	Not evaluable	PD	SD
43	Not evaluable	Not evaluable	SD	PD
44	Non-target	PD	PD	SD
45	Non-target	Non-CR/Non-PD	SD	PR
46	Non-target	Non-CR/Non-PD	SD	SD
47	Not evaluable	PD	PD	SD
48	Non-target	Non-CR/Non-PD	PD	PR
49	Target	SD	SD	PR
50	Target	SD	SD	PD
51	Non-target	Non-CR/Non-PD	PR	PD
52	Not evaluable	Not evaluable	PD	PD
53	Not evaluable	Not evaluable	PR	PR
54	Not evaluable	Not evaluable	PR	PR
55	Not evaluable	Not evaluable	PD	PD
56	Non-target	PD	PD	PD
57	Target	SD	SD	SD
58	Non-target	PD	PD	PD
59	Target	PD	PD	PD
60	Not evaluable	Not evaluable	PD	PD
61	Non-target	Non-CR/Non-PD	SD	PD

*PD* progressive disease, *SD* stable disease, *PR* partial response, *CR* complete response

Analyzing bone responders’ biological and clinical characteristics, we did not find any correlation within the univariate model (Table 5).

**Table 5:** Baseline characteristics according to immunotherapy bone-specific response (partial or complete)

	Non-responders (%)	Responders (%)	Total (%)	p value
Age				
≤ 65 years	22 (42.3)	3 (37.5)	25 (41.7)	1.000
> 65 years	30 (57.7)	5 (62.5)	35 (58.3)	
Sex				
Female	19 (35.8)	2 (25.0)	21 (34.4)	0.839
Male	34 (64.2)	6 (75.0)	40 (65.6)	
Histology				
Nonsquamous	40 (75.5)	7 (87.5)	47 (77.0)	0.762
Squamous	13 (24.5)	1 (12.5)	14 (23.0)	
Smoking status				
Former smoker	32 (62.7)	7 (87.5)	39 (66.1)	0.314
Never smoker	9 (17.6)	1 (12.5)	10 (16.9)	
Smoker	10 (19.6)		10 (16.9)	
ECOG PS				
0–1	41 (80.4)	8 (100.0)	49 (83.1)	0.386
≥ 2	10 (19.6)	0 (0.0)	10 (16.9)	
PD-L1 expression				
≥ 50%	14 (26.4)	4 (50.0)	18 (29.5)	0.310
0	12 (22.6)	1 (12.5)	13 (21.3)	
1–49%	7 (13.2)	2 (25.0)	9 (14.8)	
Unknown	20 (37.7)	1 (12.5)	21 (34.4)	
No. of metastatic sites				
≤ 2	8 (15.1)	3 (37.5)	11 (18.0)	0.297
> 2	45 (84.9)	5 (62.5)	50 (82.0)	
Type of bone met				
Mixed	13 (24.5)	3 (37.5)	16 (26.2)	0.275
Osteoblastic	13 (24.5)		13 (21.3)	
Osteolytic	27 (50.9)	5 (62.5)	32 (52.5)	
Liver met				
No	41 (77.4)	7 (87.5)	48 (78.7)	0.849
Yes	12 (22.6)	1 (12.5)	13 (21.3)	
Brain met				
No	46 (86.8)	7 (87.5)	53 (86.9)	1.000
Yes	7 (13.2)	1 (12.5)	8 (13.1)	
Line of treatment				
> 2	10 (18.9)	1 (12.5)	11 (18.0)	0.326
1	13 (24.5)	4 (50.0)	17 (27.9)	
2	30 (56.6)	3 (37.5)	33 (54.1)	
Drug				
Atezolizumab	18 (34.0)	2 (25.0)	20 (32.8)	0.610
Nivolumab	18 (34.0)	2 (25.0)	20 (32.8)	
Pembrolizumab	17 (32.1)	4 (50.0)	21 (34.4)	
Zoledronic Acid				
No	42 (80.8)	5 (71.4)	47 (79.7)	0.939
Yes	10 (19.2)	2 (28.6)	12 (20.3)	

*n.* number, *met.* Metastasis, *ECOG PS* Eastern Cooperative Oncology Group performance status

**Fig. 5:**
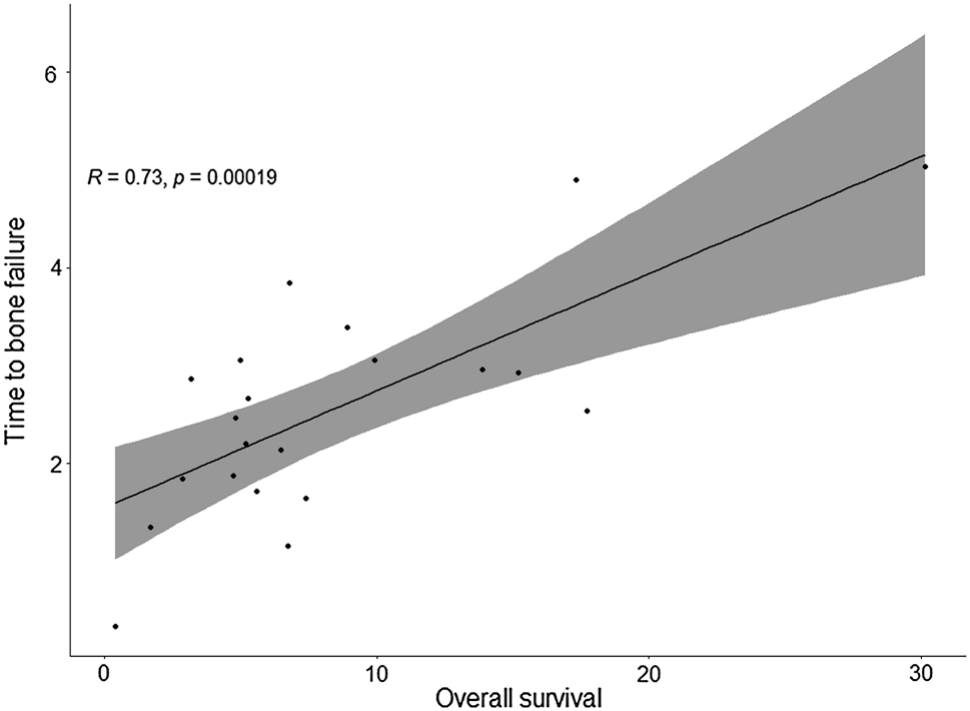
Correlation between time to bone failure (TBF) and overall survival

The median time to bone response was 2.7 months (IQR, 2.5–4.1). On the other hand, the median time to bone failure (TBF) was 2.4 months (IQR, 1.67–3.0). Considering patients with bone PD as best response to immunotherapy, we found that in 9/35 (25.7%) cases the TBF was shorter than PFS according to RECIST 1.1 criteria. The TBF was positively correlated with OS (HR = 0.73, *p* = 0.00019), as shown in (Fig. 5).

Analyzing the same bone lesions according to RECIST criteria, we found out that 10 (16.4%) were target lesions, 27 (44.3%) were considered non-target lesions, and 24 (39.3) were not evaluable due to the chosen evaluation method (CT without contrast, PET with low dose CT scan).

In addition, 12 patients (19.6%) received a palliative course of radiotherapy for pain control (single fraction, 8 Gy). No prophylactic surgery was performed.

Finally, we explored the use of bone-targeted agents. 12 patients (19.6%) received zoledronic acid, while none received denosumab. The median number of administered cycles was 6 (IQR,10.2–1). The administration of zoledronic acid was associated with a non-statistically significant prolonged median OS (7.6 vs. 4.7 months, *p* = 0.17) and PFS (4.6 vs. 2.2 months, *p* = 0.39).

## Discussion

We conducted a single-center retrospective study on 222 patients affected by advanced NSCLC, investigating the role of bone-specific response as a predictor of the efficacy of single-agent immunotherapy. Previous studies that analyzed the prognostic role of bone metastasis in NSCLC patients consistently showed decreased OS in patients with bone lesions at baseline, as compared with those without bone lesions. In a study by Qin et al. (2021), 124 out of 330 patients with metastatic NSCLC treated with immunotherapy had bone metastases at baseline, which correlated with shorter OS (5.9 months, 95% CI, 4.2–7.8) as compared to patients without bone lesions (13.4 months, 95% CI, 10.8–17.0; *p* < 0.001) (Qin et al. 2021). Similar outcomes have been reported by Kuchuk et al., as median OS in patients with or without bone metastases was 5.8 months versus 10.2 months, respectively (*p* = 0.03) (Kuchuk et al. 2015). Furthermore, a study from Li Zhang et al. showed that, among factors like histology, clinical stage, ECOG PS and serum alkaline phosphatase, the number of metastatic bone lesions also correlated with prognosis in patients treated with immune-checkpoint inhibitors. The risk of death was significantly increased in patients with multiple bone metastases compared to those with a single lesion (Odds Ratio: 2.16; 95% CI, 1.285–3.630; *p* = 0.004) (Zhang et al. 2017). Our data fit in line with these studies, showing an increased risk of death in patients with bone metastasis at diagnosis and reinforcing the internal validity of the subsequent analyses.

We then analyzed whether distinct types of bone metastases at baseline correlate with a different response to treatment. Bone metastases in patients with lung cancer are usually lytic, although mixed or osteoblastic morphologies are also observed. Distinct patterns of cytokines underlie the development of different types of lesions, according to the balance between bone formation and resorption (Wang et al. 2020). Our data showed that the type of bone metastasis does not influence the OS.

Furthermore, we assessed bone-specific responses using the MDA criteria. Our data showed that the bone-specific response assessed by MDA criteria was significantly correlated with survival outcomes. Consistently, a retrospective experience including 16 NSCLC patients treated with nivolumab evidenced that early response evaluated with MDA criteria may be a predictor of prognosis and of disease response evaluated with RECIST 1.1 criteria (Nakata et al. 2020).

The small number of patients constituted a relevant criticism of their work, impeding an affordable multivariate assessment. In addition, the authors recognized the short median follow-up time (12.2 months) as a limitation of their work. Conversely, our analysis’s median follow-up time was longer (30.1 months), probably due to the inclusion of patients treated with upfront immunotherapy.

We demonstrated that patients experiencing a bone-specific objective response (PR/CR) had longer median OS and PFS. On the other hand, a shorter TBF predicted an overall systemic treatment failure and increased risk of death, as bone PD according to MDA criteria preceded systemic disease progression in approximately 1/4 of cases. The correlation between bone-specific response and outcome has already been explored in oncogene-addicted NSCLC, and osteoblastic reactions in patients treated with EGFR inhibitors have been associated with favorable outcomes (Pluquet et al. 2010).

However, this is to our knowledge the first study that demonstrated a statistically significant correlation between bone-specific response and survival in non-oncogene addicted NSCLC patients treated with immune-checkpoint inhibitors.

It is worth noting that almost 40% of patients had bone lesions that were not evaluable with the RECIST 1.1 criteria. Notably, the response evaluation with methods different from CT scan with contrast medium, such as CT without contrast or PET with low dose CT scan, can be frequent in clinical practice. The MDA criteria can be a useful integrating tool to categorize response to therapy in these settings.

Finally, we did not find any correlation between the use of zoledronic acid and survival outcomes or bone-specific response according to MDA criteria, consistently with most studies evaluating their impact on survival in cancer patients (Henry et al. 2011; Scagliotti et al. 2012). However it should be noted that our study did not evaluate the occurrence of adverse skeletal events in relation with the use of zoledronic acid and thus the impact of this drug on prognosis has not been fully explored.

The main limitation of this work is represented by its retrospective nature and the limited sample size of patients included typically linked to a monocentric experience.

In addition, the inclusion of patients who underwent multiple lines of treatments may have affected the reliability of the analysis about the bone response, even if we preliminary considered the line of treatment within the multivariate analysis confirming the negative prognostic role of bone metastasis.

Moreover, our analysis did not explore the prognostic value of number, size or impending fracture of bone metastasis.

The investigator-related evaluation of disease progression constituted another criticism of our investigation. Nevertheless, two physicians have independently reviewed the radiological findings a posteriori. Finally, the correlation between the time to bone failure and overall survival may have been biased by the immortal time bias, albeit the median time to response and median time to bone failure were shorter than 3 months, thus considerably reducing this risk.

Overall, this might be a valid starting point for further studies analyzing the prognostic nature of bone-specific response assessed with the MDA criteria.

## Conclusion

MDA criteria represent a feasible and reliable tool in assessing bone-specific response to immunotherapy in advanced NSCLC, offering a more accurate evaluation and additional information capable of an earlier prediction of longer survival or treatment failure compared to RECIST 1.1 or iRECIST. Thus, we propose the inclusion of MDA criteria in the response assessment of future clinical trial testing immunotherapy strategies in patients with advanced NSCLC. Further studies will evaluate the consistency of our findings in NSCLC patients treated with first-line chemoimmunotherapy.

## Supplementary Information

Below is the link to the electronic supplementary material.

Supplementary Fig.1S Partial response on radiograph according to MDA criteria, corresponding to the appearance of a sclerotic rim in a previous lytic lesion of the left iliac wing (PNG 251 kb)

## Declarations

### Data availability

The datasets generated during and/or analyzed during the current study are available from the corresponding author on reasonable request.
