# Supplementary figures and images for: Bone-specific response according to MDA criteria predicts immunotherapy efficacy among advanced non-small cell lung cancer (NSCLC) patients
Source: J Cancer Res Clin Oncol. 2022 Jun 24;149(5):1835–47. doi: 10.1007/s00432-022-04120-z (PMC10097761; doi:10.1007/s00432-022-04120-z)

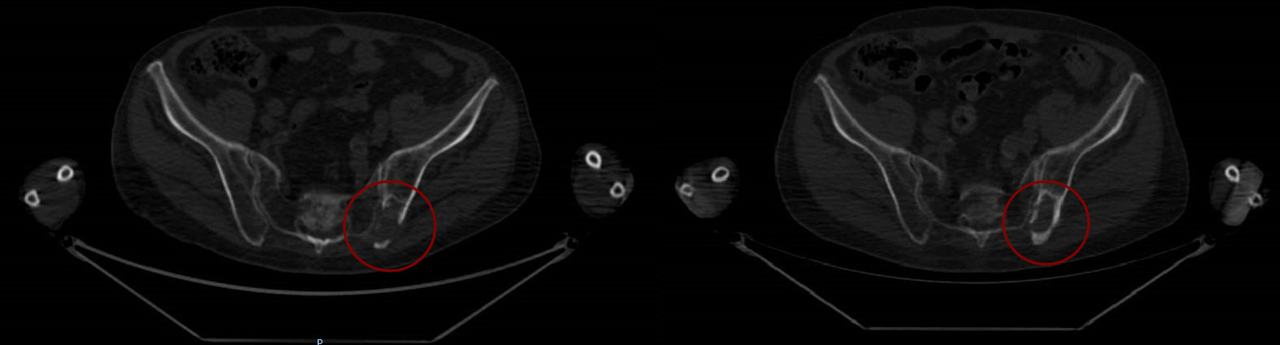

Supplement: Supplementary file 1 — Supplementary Fig.1S Partial response on radiograph according to MDA criteria, corresponding to the appearance of a sclerotic rim in a previous lytic lesion of the left iliac wing (PNG 251 kb) [file 432_2022_4120_MOESM1_ESM.png]
